# Prevalence of symptomatic polyneuropathy in patients with type 2 diabetes mellitus attending the diabetes clinic at Helen Joseph Tertiary Hospital, South Africa

**DOI:** 10.4102/safp.v67i1.6233

**Published:** 2025-12-09

**Authors:** Kaveer Thejpal, Reyna Daya, Zaheer Bayat

**Affiliations:** 1 Department of Internal Medicine, Faculty of Health Sciences, University of the Witwatersrand, Johannesburg, South Africa; 2 Department of Endocrinology and Metabolism, Faculty of Health Sciences, University of the Witwatersrand, Johannesburg, South Africa

**Keywords:** diabetes, diabetic neuropathy, distal symmetric polyneuropathy, DSPN, treatment

## Abstract

**Background:**

Diabetic neuropathy has an estimated prevalence of 50% among individuals with longstanding diabetes, with distal symmetric polyneuropathy (DSPN) being the most common manifestation. Poor glycaemic control is a recognised risk factor for DSPN. This study aimed to determine the prevalence of symptomatic DSPN in patients with type 2 diabetes mellitus (T2D) using a validated symptom-screening questionnaire – the diabetic neuropathy symptom (DNS) score. In addition, the association between haemoglobin A1c (HbA1c) and DSPN was investigated.

**Methods:**

A cross-sectional study was performed at the diabetes clinic at Helen Joseph Tertiary Hospital, Johannesburg, South Africa. A total of 206 consecutive patients with T2D were included. Underlying comorbidities and HbA1c values were obtained from patient records. The DNS score was used to assess for the presence of symptomatic DSPN.

**Results:**

The prevalence of symptomatic DSPN was 61.2%. Among those who screened positive for DSPN, 58% were not receiving pharmacological treatment for DSPN. Patients with HbA1c values of 7% – 10% and > 10% were 2.9 and 3.7 times, respectively, likely to have DSPN (prevalence ratio [PR] = 2.9; 95% confidence interval [CI] 1.5–5.4, *p* = 0.001; PR = 3.7; 95% CI 2.0–7.0, *p* < 0.001, respectively), compared with those with an HbA1c value < 7%.

**Conclusion:**

A higher than expected prevalence of symptomatic DSPN was observed in this study population, indicating the need for enhanced screening. Furthermore, a significant proportion of symptomatic patients were not receiving treatment. Poor glycaemic control with HbA1c values > 7% significantly increases the risk of DSPN.

**Contribution:**

The DNS score can easily be implemented at a primary care level to detect symptomatic neuropathy and facilitate prompt treatment.

## Introduction

Diabetes mellitus (DM) is a metabolic disorder that results in chronic hyperglycaemia, together with impaired metabolism of macronutrients – carbohydrates, fats and proteins.^1,2^ Type 2 diabetes mellitus (T2D) – which occurs because of a combination of insulin resistance and a progressive loss of sufficient insulin secretion – represents the majority of DM cases, accounting for more than 90% of all diabetes cases.^1,2,3^

The prevalence of DM has been rapidly increasing over the last decade, both globally and in South Africa. Recent data estimate that there are currently 537 million people with DM worldwide, and this is projected to increase to 783 million people by 2045.^4^ In South Africa, the current prevalence of DM (among the adult population aged 20–79 years) is estimated to be around 10.8%.^4^

The increasing prevalence of T2D can be attributed to a number of different factors, including the ageing of the general population, as well as greater urbanisation of the population, which in turn contributes to increased obesity driven by sedentary lifestyles and unhealthy dietary consumption.^2,4^

There are numerous complications associated with DM, which can be broadly categorised as microvascular or macrovascular. Microvascular complications include retinopathy, neuropathy and nephropathy.^2,5,6^ Macrovascular complications include coronary heart disease (CHD), cerebrovascular disease (CVD) and peripheral arterial disease (PAD).^2,5,6^ Microvascular complications of DM have been shown to have a higher prevalence than the macrovascular complications.^6,7^

Diabetic neuropathy is a common complication of DM and is estimated to affect approximately 50% of people with longstanding DM.^2^ Neuropathy because of DM can occur in various forms, such as distal symmetric polyneuropathy (DSPN) (which is often synonymously referred to as ‘peripheral neuropathy’), autonomic neuropathy, mononeuropathy, radiculopathy or mononeuritis multiplex.^8^ Distal symmetric polyneuropathy is by far the most common manifestation, representing around 75% of diabetic neuropathy cases.^8^

Distal symmetric polyneuropathy can occur in both type 1 diabetes mellitus (T1D) and T2D; however, there are significant differences in its prevalence. In T1D, it is estimated that DSPN will occur in 20% of individuals after 20 years duration.^8^ In T2D, up to 15% of patients may have DSPN at the time of initial diagnosis and this rate increases drastically to approximately 50% of patients after 10 years.^8^

The level of glycaemic control – typically assessed using haemoglobin A1c (HbA1c) measurements – and the duration of DM are the most significant risk factors for the development of DSPN.^2,9^ This is particularly demonstrated in T1D, while in T2D, the condition is driven by multifactorial metabolic risk factors.^2,9^ Local guidelines generally aim to achieve an HbA1c value < 7% to prevent microvascular complications of DM.^1^ Furthermore, other factors such as hypertension, smoking, dyslipidaemia and increased body mass index (BMI) are also strongly associated with the development of DSPN.^2,6,8^

Screening for DSPN is an essential component in the care of patients with diabetes, as it allows for early detection of neuropathy, treatment of symptoms and the implementation of strategies to prevent debilitating complications such as foot ulceration, Charcot neuropathic arthropathy and falls and fractures.^8,9^ Distal symmetric polyneuropathy is predominantly a clinical diagnosis, based on a combination of history and examination findings, and it is recommended that only those with atypical presentations or diagnostic uncertainty be referred for further electrophysiological nerve testing.^8^

Current guidelines recommend that all individuals with T2D should be screened for DSPN at the time of diagnosis, and thereafter on an annual basis.^8^ Screening for DSPN is carried out either by questionnaire or by clinical examination (10 g monofilament, tuning fork or Ipswich Touch Test).^1,8^ There have been various scoring systems or questionnaires developed for diabetic neuropathy, with the diabetic neuropathy symptom (DNS) score by Meijer et al. being an example of a validated symptom-scoring questionnaire for diabetic polyneuropathy, consisting of only four questions.^10^ In addition, the DNS score requires no equipment and can easily be performed in an outpatient clinic or primary care setting.

There is currently a paucity of research regarding the prevalence of diabetic neuropathy in the South African context.

### Objectives

The primary objective of this study was to determine the prevalence of symptomatic polyneuropathy (DSPN) in patients with type 2 diabetes mellitus attending the diabetes clinic at Helen Joseph Tertiary Hospital, using a validated screening questionnaire.

The secondary objective was to investigate the association between glycaemic control, as assessed by HbA1c levels, and symptomatic DSPN in patients with type 2 diabetes mellitus.

## Research methods and design

### Study design and population

This cross-sectional study was performed at Helen Joseph Tertiary Hospital (HJH) – an academic hospital in Johannesburg, South Africa, affiliated to the University of the Witwatersrand (Wits) and serving a population of approximately 1 million people. Patient recruitment and data collection were performed in a prospective manner, using a consecutive sampling method, over a 3-month period.

All patients living with T2D who attended the outpatient diabetes clinic at HJH, between 01 June 2023 and 31 August 2023, were considered for inclusion in the study. This clinic has an annual patient volume of approximately 4000. Patients below the age of 18 years, pregnant women, patients with human immunodeficiency virus (HIV) infection and/or hypothyroidism, and those with neuropathy because of other non-diabetic causes, for example, vitamin B12 deficiency, were excluded from the analysis. In addition, patients who declined to participate in the study were excluded.

### Sample size

The sample size needed for this study was calculated based on a 50% prevalence ±5%, with a 10% precision, assuming an *α*-level of 5% and 80% power.^8^ Based on these parameters, a minimum sample size of 195 patients was required. By the end of the study period, data from a total of 206 patients were obtained for analysis.

### Data collection

The principal investigator interviewed each patient individually at the diabetes clinic and physically captured patient information and responses to the questionnaire on a pre-printed data collection sheet. Each patient was assigned a unique study number to ensure anonymity of the data. A confidential list was kept, accessible only to the principal investigator, linking the study number to the patient file number. This was used to prevent duplicate entries.

The data collection sheet consisted of two sections. The first section contained demographic information (age, gender, race), body measurements (weight, height, BMI), known comorbidities (hypertension, dyslipidaemia, chronic kidney disease, peripheral vascular disease and current smoking status), and recent HbA1c blood results. The most recent HbA1c results from the preceding 6 months were used for analysis, in accordance with the clinic protocol aimed at cost-effective testing and adherence to institutional austerity measures. In addition, it was also recorded if a patient was currently receiving any pharmacological medication to treat neuropathy symptoms, as per current recommendations, for example, tricyclic antidepressants, pregabalin etc.^1^ All the required information and blood tests that are routinely performed during diabetes clinic visits were available from patients’ records.

**FIGURE 1:**
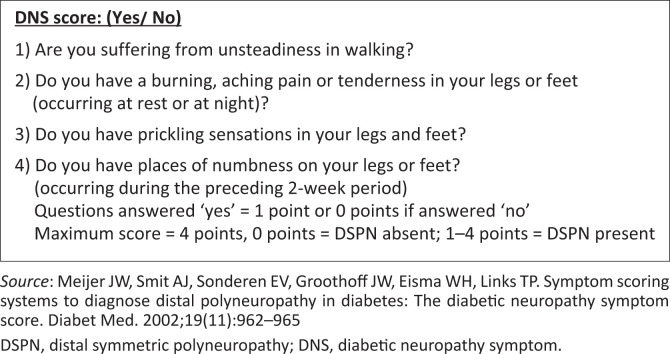
Diabetic neuropathy symptom score.

The second section of the data collection sheet contained the DNS score questionnaire – consisting of four (‘yes’ or ‘no’) questions (Figure 1).^10^ This is a validated symptom-scoring questionnaire for diabetic polyneuropathy, with a sensitivity of 79% and specificity of 78%.^10^ The DNS score was much quicker to perform than other scoring systems and required no specific equipment, making it suitable to implement in an outpatient clinic in both primary care and specialist centres. Permission was obtained from the author prior to using the questionnaire for this study.

The DNS score was administered verbally by the principal investigator in English. Given the simplicity of the questions, most participants were able to understand and respond in English without difficulty. Where necessary, multilingual clinic nurses assisted with translation of the questions to ensure participants’ comprehension.

### Data management and analysis

The information from physical data collection sheets was transcribed into a Microsoft Excel spreadsheet (Microsoft Corporation, version 2021). The data were then exported into Stata version 18.5 (Stata Corporation, College Station, Texas, United States) for analysis. Descriptive statistics – medians and interquartile ranges for continuous variables and frequencies and percentages for categorical data – were used to describe the enrolled patients.

The prevalence of DSPN was determined as the number of patients who had scores 1–4 divided by the number of patients enrolled and expressed as a percentage. The prevalence of DSPN was described by different age categories, gender, BMI and categories of comorbidities. Univariable and multivariable Poisson regression with robust error variance was used to determine the factors independently associated with DSPN in this population. Variables with *p*-values < 0.2 in univariable analyses were included in the multivariable models. The extent of association between independent variables and prevalence of DSPN was determined as prevalence ratio (PR) and presented as estimates with 95% confidence intervals around them.

### Ethical considerations

Ethical clearance to conduct the study was granted by the University of the Witwatersrand HREC (Human Research Ethics Committee) on 11 July 2023. The ethical clearance number is M230260. Permission was also obtained from the CEO of HJH. In addition, each participant was given a study information sheet, and they provided informed consent to participate in the study.

## Results

### Population demographics

Data from 215 patients were captured for the study. Nine patients were excluded from the analysis because of incomplete or missing data. Therefore, a total of 206 patients were included in the final analysis, consisting of 117 (56.8%) female patients and 89 (43.2%) male patients. The majority of the patients (72.8%, *n* = 150) were 50 years or older. Black African individuals made up the majority of the study population (56.3%, *n* = 116), followed by mixed race (21.4%, *n* = 44), white (15.5%, *n* = 32), and Indian (6.8%, *n* = 14). The median HbA1c value for the study population was 8.6%. The complete demographics of the study population are summarised in Table 1.

**TABLE 1:** Characteristics of the study population.

Variables	*n*	%	Median	IQR
**Demographic characteristics**				
Age (years)	-	-	57.00	49–64
< 50	56	27.2	-	-
50–69	127	61.6	-	-
≥ 70	23	11.2	-	-
Gender				
Female	117	56.8	-	-
Male	89	43.2	-	-
Race				
Black African people	116	56.3	-	-
Mixed race	44	21.4	-	-
Indian people	14	6.8	-	-
White people	32	15.5	-	-
**Clinical characteristics**				
Weight in kg	-	-	82.20	72–95
Height in meters	-	-	1.66	1.60–1.72
BMI in kg/m^2^	-	-	29.60	26.0–33.8
**BMI category**				
< 25	39	18.9	-	-
25–29	72	35.0	-	-
≥ 30	95	46.1	-	-
HbA1c (%)	-	-	8.60	7.1–10.9
**HbA1c category (%)**				
< 7	43	20.9	-	-
7–10	92	44.7	-	-
> 10	71	34.1	-	-

BMI, body mass index; IQR, interquartile range; HbA1c, haemoglobin A1c.

**FIGURE 2:**
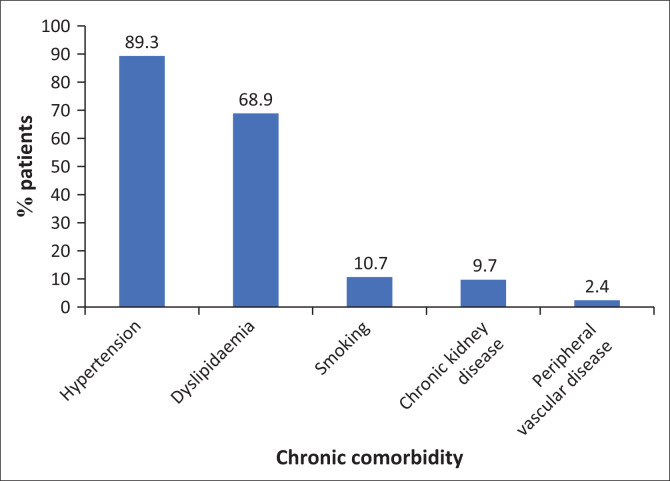
Distribution of chronic comorbidities in the study population (*N* = 206).

The known chronic comorbidities of the study population were also captured for analysis. The most common being hypertension (89.3%), followed by dyslipidaemia (68.9%), current smoking (10.7%), chronic kidney disease (9.7%) and peripheral vascular disease (2.4%) (Figure 2).

### Diabetic neuropathy symptom score

The presence of symptomatic DSPN among the study population was assessed by means of the DNS score – a validated symptom-scoring questionnaire for diabetic polyneuropathy.^10^ A score of 0 points meant that peripheral neuropathy was absent, while a score of 1–4 points meant that symptomatic peripheral neuropathy was present.

**FIGURE 3:**
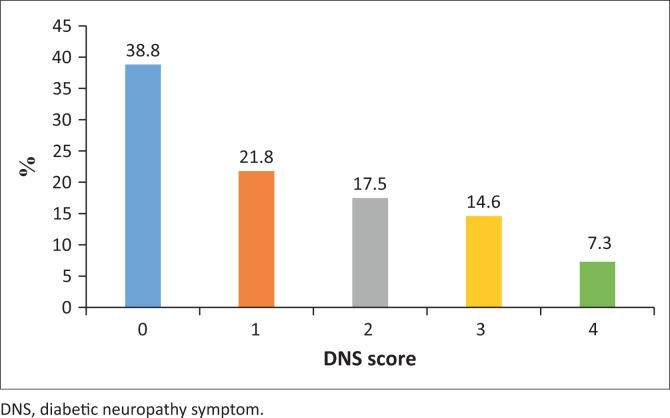
Distribution of diabetic neuropathy symptom scores among patients with type 2 diabetes mellitus attending the diabetes clinic at Helen Joseph Hospital between 01 June 2023 and 31 August 2023 (*N* = 206).

Out of the total study population (*n* = 206), symptomatic DSPN was present in 126 patients (61.2%), with 7.3% of patients answering ‘yes’ to all four symptom-screening questions. The proportion of patients with no symptoms of DSPN (score of 0) was 38.8%. The distribution of DNS scores is depicted in Figure 3.

### Treatment for distal symmetric polyneuropathy

**FIGURE 4:**
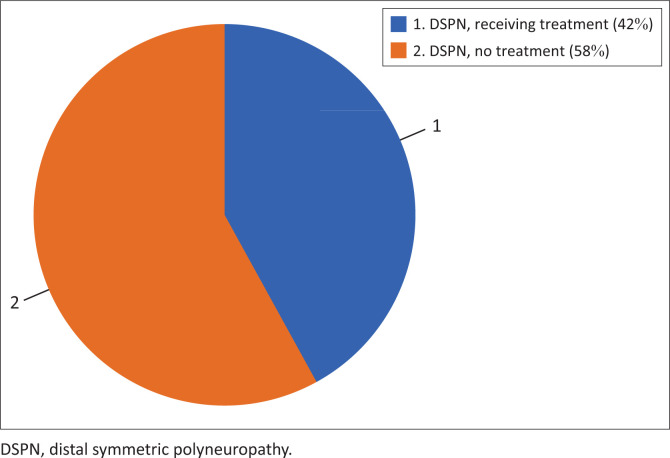
Distal symmetric polyneuropathy pharmacological treatment status (*N* = 126).

Patients who screened positive for DSPN were also categorised based on their current pharmacological treatment status – that is, whether or not they were currently receiving any recommended pharmacological medication to treat neuropathy symptoms – as per current guidelines.^1^ Out of the 126 patients who screened positive for DSPN (DNS score of 1–4), 73 (58%) patients were not taking any medication to manage DSPN. Figure 4 summarises the treatment distribution.

### Distal symmetric polyneuropathy prevalence

Out of the total study population of 206 patients, 126 patients had screened positive for symptomatic DSPN based on the DNS score, equating to a DSPN prevalence of 61.2%. The prevalence of DSPN for the demographics, clinical characteristics and chronic comorbidities of the study population was assessed and summarised in Table 2.

**TABLE 2:** Distal symmetric polyneuropathy prevalence among the study population.

Category	*n*	*N*	%	95% CI	*Χ*^*2*^ *p*
Overall prevalence	126	206	61.2	54.3–67.6	-
**Age (years)**	-	-	-	-	< 0.001
< 50	19	56	33.9	22.7–47.3	-
50–69	91	127	71.7	63.2–78.8	-
≥ 70	16	23	69.6	48.3–84.8	-
**Gender**	-	-	-	-	0.024
Female	79	117	67.5	58.5–75.4	-
Male	47	89	52.8	42.4–63.0	-
**Race**	-	-	-	-	< 0.001
Black African people	55	116	47.4	38.5–56.5	-
Mixed race people	34	44	77.3	62.6–87.4	-
Indian people	10	14	71.4	43.8–88.9	-
White people	27	32	84.4	67.4–93.4	-
**BMI**	-	-	-	-	0.370
< 25	20	39	51.3	35.9–66.4	-
25–29	46	72	63.9	52.2–74.2	-
≥ 30	60	95	63.2	53.0–72.3	-
**HbA1c (%)**	-	-	-	-	< 0.001
< 7	8	43	18.6	9.5–33.1	-
7 – 10	62	92	67.4	57.1–76.2	-
> 10	56	71	78.9	67.8–86.9	-
**Hypertension**	-	-	-	-	0.039
No	9	22	40.9	22.7–61.9	-
Yes	117	184	63.6	56.4–70.3	-
**Dyslipidaemia**	-	-	-	-	0.027
No	32	64	50.0	37.9–62.1	-
Yes	94	142	66.2	58.0–73.5	-
**Smoking**	-	-	-	-	0.239
No	110	184	59.8	52.5–66.7	-
Yes	16	22	72.7	50.9–87.3	-
**Chronic kidney disease**	-	-	-	-	0.910
No	114	186	61.3	54.1–68.1	-
Yes	12	20	60.0	37.9–78.7	-
**Peripheral vascular disease**	-	-	-	-	0.159*
No	121	201	60.2	53.2–66.8	-
Yes	5	5	100.0	-	-

BMI, body mass index; CI, confidence interval; χ^2^, Chi-squared; HbA1c, haemoglobin A1c; DSPN, distal symmetric polyneuropathy; *n*, number with DSPN; *N*, total.

* , Fischer’s exact *p*-value.

Distal symmetric polyneuropathy prevalence increased significantly with age and was present in 71.7% and 69.6% of patients aged 50–69 and ≥ 70 years, respectively, (95% CI 63.2–78.8 and 48.3–84.8) compared with 33.9% in those aged < 50 years (95% CI 22.7–47.3; *p* < 0.001). A statistically significant higher prevalence of DSPN was observed in female patients compared with male patients (67.5% vs. 52.8%, respectively; 95% CI 58.5–75.4; vs. 42.2–63.0; *p* = 0.02). White patients had the highest DSPN prevalence (84.4%; 95% CI 67.4–93.4), followed by mixed race (77.3%; 95% CI 62.6–87.4), Indian (71.4%; 95% CI 43.8–88.9) and African (47.4%; 95% CI 38.5–56.5) patients. No significant difference in DSPN prevalence was observed across BMI categories on univariable analysis (*p* = 0.37).

A significant increase in DSPN prevalence was observed with higher HbA1c levels: 67.4% (95% CI 57.1–76.2) in those with an HbA1c level of 7% – 10% and 78.9% (95% CI 67.8–86.9) in those with an HbA1c level > 10%, compared with 18.6% (95% CI 9.5–33.1) in those with an HbA1c level < 7% (*p* < 0.001).

With regard to comorbidities among the study population, a statistically significant increased prevalence of DSPN was observed among patients with hypertension (63.6%; 95% CI 56.4–70.3, *p* = 0.03) and dyslipidaemia (66.2%; 95% CI 58.0–73.5, *p* = 0.02). No significant difference in DSPN prevalence was found among patients with chronic kidney disease (*p* = 0.91), peripheral vascular disease (*p* = 0.15) and those who smoked (*p* = 0.23).

The strength of association of each population characteristic with DSPN prevalence was determined using univariable and multivariable Poisson regression and presented as a PR. Variables with *p*-values < 0.2 in univariable analysis were included in the multivariable analysis (Table 3).

**TABLE 3:** Prevalence ratios for distal symmetric polyneuropathy in the study population.

Variable	Univariable			Multivariable		
	PR	95% CI	*p*	PR	95% CI	*p*
**Age (years)**						
< 50	1	-	-	1	-	-
50–69	2.11	1.44–3.10	< 0.001	1.56	1.08–2.24	0.017
≥ 70	2.05	1.30–3.23	0.002	1.45	0.90–2.33	0.128
**Gender**						
Female	1.28	1.01–1.62	0.039	1.24	1.00–1.53	0.046
Male	1	-	-	1	-	-
**Race**						
Black African people	1	-	-	1	-	-
Mixed race people	1.63	1.27–2.09	< 0.001	1.22	0.96–1.55	0.101
Indian people	1.51	1.03–2.21	0.003	1.18	0.80–.74)	0.412
White people	1.78	1.40–2.27	< 0.001	1.25	1.01–1.58	0.058
**BMI**						
< 25	1	-				
25–29	1.25	0.88–1.77	0.221	-	-	-
≥ 30	1.23	0.87–1.73	0.233	-	-	-
**HbA1c (%)**						
< 7	1	-	-	1	-	-
7–10	3.62	1.90–6.89	< 0.001	2.88	1.53–5.42	0.001
> 10	4.24	2.24–8.03	< 0.001	3.74	1.99–7.04	< 0.001
**Hypertension**						
No	1	-	-	1	-	-
Yes	1.55	0.93–2.60	0.093	1.10	0.71–1.73	0.665
**Dyslipidaemia**						
No	1	-	-	1	-	-
Yes	1.32	1.01–1.74	0.043	1.18	0.94–1.49	0.146
**Smoking**						
No	1	-	-	1	-	-
Yes	1.22	0.92–1.61	0.173	1.12	0.92–1.37	0.262
**Chronic kidney disease**						
No	1	-	-	-	-	-
Yes	1.02	0.70–1.49	0.912	-	-	-
**Peripheral vascular disease**						
No	1	-	-	1	-	-
Yes	1.66	1.48–1.86	< 0.001	1.65	1.23–2.22	0.001

PR, prevalence ratio; BMI, body mass index; CI, confidence interval; HbA1c, haemoglobin A1c.

In terms of age, patients in the 50–69 and ≥ 70 age categories were 2.1 and 2.0 times more likely, respectively, to have DSPN compared with those aged < 50 years (95% CI 1.4–3.1 and 1.3–3.2). On multivariable analysis, age 50–69 years remained independently associated with DSPN (adjusted PR 1.5; 95% CI 1.0–2.2; *p* = 0.017). Female patients were 1.2 times more likely to have DSPN compared with male patients (95% CI 1.0–1.6), and this remained a significant association on multivariable analysis (adjusted PR 1.2; 95% CI 1.0–1.5; *p* = 0.046).

Compared with African individuals, white, mixed race and Indian individuals were significantly more likely to have DSPN, with PRs of 1.7 (95% CI 1.4–2.2), 1.6 (95% CI 1.2–2.0), 1.5 (95% CI 1.0–2.2), respectively. However, these associations were not significant on multivariable analysis. No significant difference in DSPN prevalence was found among the BMI categories.

Patients with HbA1c values of 7% – 10% and > 10% were 3.6 (95% CI 1.9–6.8) and 4.2 (95% CI 2.2–8.0) times more likely, respectively, to have DSPN than those with an HbA1c value < 7%. This association remained significant on multivariable analysis, with adjusted PRs of 2.8 (95% CI 1.5–5.4; *p* = 0.001) and 3.7 (95% CI 1.9–7.0; *p* < 0.001) for HbA1c values of 7% – 10% and > 10%, respectively.

Among the chronic comorbidities of the study population, no significant associations with DSPN were found for hypertension (*p* = 0.093), current smoking (*p* = 0.173), or chronic kidney disease (*p* = 0.912) on univariable analysis. Those with dyslipidaemia and peripheral vascular disease were 1.3 (95% CI 1.0–1.7) and 1.6 (95% CI 1.4–1.8) times more likely, respectively, to have DSPN. However, on multivariable analysis, only peripheral vascular disease remained significantly associated with DSPN (adjusted PR 1.6; 95% CI 1.23–2.22; *p* = 0.001).

## Discussion

### Diabetic Neuropathy Symptom score

The DNS score was used in this study to determine the prevalence of symptomatic DSPN. This scoring system was selected because of its concise design and ease of use, consisting of only four (‘yes’ or ‘no’) questions. Despite its simplicity, when compared with the more extensive and widely used neuropathy symptom score (NSS), which consists of 17 questions, the DNS score showed a high correlation between scores (Spearman *r* = 0.88) and good reproducibility (Cohen weighted *κ* = 0.78–0.95).^10^

As per current recommendations, screening for neuropathy in patients with T2D should occur at the time of diagnosis and, thereafter, at least annually.^1,8^ The Society for Endocrinology, Metabolism and Diabetes of South Africa (SEMDSA) guidelines recommend that neuropathy screening should occur using clinical methods such as a 10g monofilament examination, 128Hz tuning fork, or Ipswich touch test.^1^ These clinical modalities all have the advantage of detecting sensory deficits, which may occur in otherwise asymptomatic cases of neuropathy.

In the validation study by Meijer et al., the DNS score correlated weakly with monofilament testing (Spearman *r* = 0.25; *p* < 0.05).^10^ This weak correlation suggests that these tests assess different aspects of neuropathy, with the DNS score detecting the symptomatic features, while clinical methods such as monofilament testing detect objective sensory loss. In practice, patients may be symptomatic for DSPN without sensory loss on objective examination, or vice versa, and therefore highlights that symptom screening tools such as the DNS score and objective clinical assessment are complementary to each other and should be used in conjunction for comprehensive neuropathy assessment.

In this context, the concise design of the DNS score makes it particularly suitable for high-volume outpatient clinic settings that face time and resource constraints, allowing for rapid identification of patients with symptomatic DSPN. As no standardised template for symptom screening currently exists in local guidelines, this study provides evidence for incorporating the DNS score into the routine clinical assessments that are already recommended for neuropathy screening.

### Key findings

In this study, the prevalence of DSPN among patients with T2D was found to be 61.2%, which is significantly higher than the estimated prevalence of 50% in the current literature.^8^ There were 126 (out of a total of 206) individuals who met the criteria for DSPN. Given this sample size, post hoc power calculation showed that the achieved sample size had 97.7% power to determine a DSPN prevalence of 61.2% ± 6.5%, assuming *α* = 0.05. The higher-than-expected prevalence of DSPN observed in this study may be attributed to the tertiary-care setting in which this study was conducted, which predominantly includes patients with more advanced disease, suboptimal glycaemic control and multiple metabolic comorbidities. Similarly high prevalence estimates have been reported in other hospital-based studies across Africa, including a Tanzanian cohort reporting a 72% prevalence among patients attending a tertiary diabetes clinic.^11^

After determining DSPN prevalence, the DNS scores were then analysed in relation to neuropathy treatment status (Figure 4). Of the 126 patients who had DSPN symptoms (DNS score ≥ 1), more than half (58%) were not receiving any pharmacological treatment for their symptoms. This finding likely reflects the under-detection of neuropathic symptoms because of the absence of standardised symptom-screening tools in current practice, combined with time constraints and high patient volumes commonly encountered in outpatient clinics.

This highlights the utility of a concise symptom-screening tool such as the DNS, which can easily be incorporated into routine clinical assessments to allow for more regular neuropathy screening without imposing additional time burden. In addition, this underscores the need for healthcare providers to remain familiar with current guideline-recommended treatment options for symptomatic DSPN, including tricyclic antidepressants (e.g. amitriptyline), serotonin-noradrenaline reuptake inhibitors (e.g. duloxetine) and anticonvulsants (e.g. pregabalin).^1,8^

### Distal symmetric polyneuropathy associations

The PR was used to reflect the association between the study population characteristics and DSPN. Univariable and multivariable Poisson regression were used to determine the PR, as this method has been shown to be a better alternative to logistic regression when determining metabolic syndrome associations.^12^

According to current literature, increasing age is recognised as a non-modifiable risk factor for diabetic neuropathy.^13^ In this study, DSPN prevalence increased significantly with age, with individuals between ages 50 and 69 years being 1.5 times more likely to have DSPN compared with those aged < 50 years (95% CI 1.0–2.2; *p* = 0.017). This finding supports existing evidence, demonstrating an association between advancing age and higher DSPN prevalence. The absence of a significant association in individuals aged ≥ 70 years is inconsistent with this trend and may be attributed to the smaller sample size in this age category (*n* = 23), which likely reduced the statistical power to detect an independent association on multivariable analysis.

Current literature does not identify gender as an independent risk factor for diabetic neuropathy.^9,13^ In this study, however, female patients were 1.2 times more likely to have DSPN compared with male patients (95% CI 1.0–1.5; *p* = 0.046). The significant association observed in this cohort may be explained by population differences, as female patients comprised a larger proportion of the study population (117 vs. 89 males), as well as by potential gender differences in subjective symptom perception and reporting.

According to current literature, BMI is known to be associated with the development of DSPN.^2,6,8^ However, in this study, no significant association of BMI with DSPN was observed in univariable analysis. This could be because of the restricted range of BMI in this study population, with 81.1% of patients being in the overweight or obese categories (BMI ≥ 25 kg/m^2^), with a limited representation of those in the normal BMI range (< 25kg/m^2^), thereby reducing the statistical power of this study to detect an association with DSPN.

Poor glycaemic control is well recognised as one of the most significant risk factors for the development of DSPN, particularly in T1D.^2,8^ In T2D, however, glycaemic control alone has been shown to be insufficient to prevent diabetic neuropathy, as concomitant metabolic abnormalities such as obesity and dyslipidaemia also contribute to its pathogenesis.^9,13^ This underscores the need for a holistic approach to neuropathy prevention that addresses all associated metabolic risk factors.

The mechanism of neuronal injury in chronic hyperglycaemia is well established and involves oxidative stress induced by excess glycolysis, leading to the overproduction of reactive oxygen species. In addition, the formation of advanced glycation end products (AGEs) further impairs protein structure and cellular function.^9^

This study also sought to evaluate the effect of chronic hyperglycaemia, as assessed by HbA1c measurements, to determine whether an association with DSPN was present. Current guidelines recommend maintaining HbA1c values < 7% to prevent diabetic complications such as DSPN.^1^ In this study, rising HbA1c values were significantly associated with increased DSPN prevalence. Patients with HbA1c values 7% – 10% and > 10% were 2.8 (95% CI 1.5–5.4; *p* = 0.001) and 3.7 (95% CI 1.9–7.0; *p* < 0.001) times more likely, respectively, to have DSPN, compared with those with an HbA1c value < 7%. These findings confirm and highlight the strong association between poor glycaemic control and the development of DSPN.

### Chronic comorbidities

When the chronic comorbidities of the study population were analysed, peripheral vascular disease demonstrated a statistically significant association with DSPN. On multivariable analysis, affected individuals were 1.6 times more likely to have DSPN (95% CI 1.23–2.22; *p* = 0.001), consistent with the current literature identifying vascular disease as a risk factor for diabetic neuropathy.^14^

The association between abnormal lipid profiles and diabetic neuropathy has been demonstrated in the literature, with elevated triglyceride and low-density lipoprotein (LDL) cholesterol levels shown to increase the risk of diabetic neuropathy in a recent meta-analysis.^15^ In this study, dyslipidaemia appeared to be associated with DSPN on univariable analysis (PR = 1.32, *p* = 0.043); however, no statistically significant increase in prevalence of DSPN was observed in multivariable analysis (*p* = 0.146). It is important to notice that quantitative lipid measurements were not recorded in this study, and analysis was limited to the presence or absence of dyslipidaemia, which may limit the assessment of potential associations with DSPN.

Hypertension and smoking, which were known to be associated with DSPN in the current literature, showed no significant association with DSPN in this study.^2,6,8^ This may be attributed to the limited categorisation of these variables during data collection and analysis, as smoking was recorded only as a binary variable (current vs. non-smoker) without quantifying exposure in pack-years, and hypertension was not categorised according to duration or level of control. As a result, this may have reduced the study’s ability to detect dose-dependent or severity-related associations for these risk factors.

Although chronic kidney disease has been demonstrated to be associated with DSPN development in some studies, no significant association was observed in this study population.^16^ However, the specific stage of kidney disease and glomerular filtration rate (GFR) for each patient were not recorded, which may explain the absence of a detectable association.

### Limitations

Despite this study being strongly powered to determine DSPN prevalence, there were certain limitations. Firstly, this was a cross-sectional study, which limits the ability to establish a causal relationship between T2D and DSPN. Secondly, this was a single-centre study conducted in a tertiary hospital, which may limit the generalisability of the findings to other patient populations. Thirdly, the DNS score relies on patient-reported symptoms to detect DSPN; this allows for potential reporting bias and does not account for asymptomatic individuals with DSPN. Fourthly, the duration of T2D was not assessed in this study, which may have confounded the observed association between HbA1c and DSPN.

Lastly, there is a possibility that patients with non-diabetic causes of neuropathy were included in the study. Current guidelines recommend that other common causes of neuropathy be excluded before diagnosing DSPN, such as vitamin B12 deficiency and thyroid disease, both of which were part of the exclusion criteria for the study.^8^ However, the limitation exists as it is not feasible to test for and exclude all other non-diabetic causes of neuropathy.

### Implications and recommendations

The high prevalence of symptomatic DSPN observed in this study, coupled with the finding that more than half of affected patients were not receiving targeted pharmacological treatment for neuropathic symptoms, highlights an important and potentially correctable gap in current diabetes care.

While existing local guidelines provide recommendations for clinical neuropathy screening assessment – such as the 10g monofilament examination or Ipswich touch test – they do not provide a structured symptom assessment method to complement clinical screening.^1^ The DNS score offers a rapid and easily implementable screening option that can be integrated into routine chronic-care reviews of patients within primary care and family medicine settings. Its simplicity also makes it well-suited for use in nurse-led screening initiatives in primary care clinics, potentially enabling earlier identification and management of symptomatic neuropathy.

Incorporation of a structured screening tool such as the DNS into current SEMDSA and Department of Health guidelines may strengthen the comprehensiveness of diabetes care across all levels of the health system. Future research should focus on evaluating the implementation of the DNS score across multiple centres – including rural and urban primary care clinics – and correlating its findings with objective measures of neuropathy to further validate its role in neuropathy screening.

## Conclusion

Diabetic neuropathy is a common complication in patients with T2D and can result in significant morbidity and complications. This study demonstrated a higher-than-expected prevalence of DSPN (61.2%) compared with previous estimates, highlighting the need for healthcare providers to prioritise regular neuropathy screening as part of routine diabetes care.

Furthermore, a significant proportion of symptomatic patients (58%) were not receiving appropriate pharmacological treatment, emphasising the need to improve identification of symptomatic patients and facilitate access to guideline-based therapy.

The DNS score, because of its ease of use and efficient design, provides an easily implementable symptom-screening tool that can be implemented at a primary healthcare level and allows for the detection of neuropathy symptoms outside of specialist settings. Its integration into routine clinical reviews may enable earlier detection and implementation of therapeutic interventions for neuropathy, potentially preventing progression and reducing neuropathy-related complications.

The association between glycaemic control, measured by HbA1c values, and DSPN was reaffirmed, with this study demonstrating significantly higher prevalence rates among patients with HbA1c values > 7%. These findings align with current recommendations maintaining an HbA1c level < 7% in individuals with T2D and emphasise its importance in the holistic metabolic management of these patients within primary care settings.
